# Turbulence accelerates the growth of drinking water biofilms

**DOI:** 10.1007/s00449-018-1909-0

**Published:** 2018-02-10

**Authors:** E. Tsagkari, W. T. Sloan

**Affiliations:** College of Science and Engineering, School of Engineering, University of Glasgow, Glasgow, G12 8LT UK

**Keywords:** Biofilms, Drinking water, Flow regimes, Reactor

## Abstract

Biofilms are found at the inner surfaces of drinking water pipes and, therefore, it is essential to understand biofilm processes to control their formation. Hydrodynamics play a crucial role in shaping biofilms. Thus, knowing how biofilms form, develop and disperse under different flow conditions is critical in the successful management of these systems. Here, the development of biofilms after 4 weeks, the initial formation of biofilms within 10 h and finally, the response of already established biofilms within 24-h intervals in which the flow regime was changed, were studied using a rotating annular reactor under three different flow regimes: turbulent, transition and laminar. Using fluorescence microscopy, information about the number of microcolonies on the reactor slides, the surface area of biofilms and of extracellular polymeric substances and the biofilm structures was acquired. Gravimetric measurements were conducted to characterise the thickness and density of biofilms, and spatial statistics were used to characterise the heterogeneity and spatial correlation of biofilm structures. Contrary to the prevailing view, it was shown that turbulent flow did not correlate with a reduction in biofilms; turbulence was found to enhance both the initial formation and the development of biofilms on the accessible surfaces. Additionally, after 24-h changes of the flow regime it was indicated that biofilms responded to the quick changes of the flow regime. Overall, this work suggests that different flow conditions can cause substantial changes in biofilm morphology and growth and specifically that turbulent flow can accelerate biofilm growth in drinking water.

## Introduction

The provision of safe drinking water is a top priority in all societies. Managing biofilms that are formed on the inside surface of drinking water pipes is a concern of water utilities around the world as these biofilms can affect the aesthetics of drinking water [1–3]. Hydrodynamics exert a significant influence on biofilms [4–9] and in particular on the spatial distribution of bacteria [10]. Therefore, this study was set up to look at how biofilms grow under three distinct flow regimes: turbulent, transition and laminar flow.

There is a wealth of literature that supports the fact that biofilm structure is intimately linked with hydrodynamics [11–15]. These studies have been mostly focused on the growth and detachment of bacteria under a constant flow regime. In laminar flows, biofilms are found to create patchy structures [13], whereas in turbulent flows, biofilms are found to create elongated streamers [16]. The formation of streamers has been suggested to cause firmer adhesion of bacteria to the available surfaces and promotion of microcolonies formation. Streamers may consist mainly of extracellular polymeric substances (EPS) and are found to improve the resistance of the biofilm to the external shear [6]. The structure of biofilms may impact biofilm development, mass transfer processes, oxygen distribution and frictional resistance in pipelines [17–19] and thus, it is important for researchers to extend the existing knowledge.

Hydrodynamics are found to affect biofilm thickness and density. High detachment forces, caused by increased shear stresses, have been shown to lead to denser biofilms that are mechanically more stable [4, 20]. As far as the effect of hydrodynamics on biofilm thickness is concerned, there is limited knowledge regarding biofilms in drinking water systems. The prevailing view is that biofilm development is hindered by higher shear stresses due to higher detachment forces, which are applied to the biofilm [6, 11, 21]. However, turbulence has been also found to promote the development of thick biofilms [4] probably due to the increased transport of nutrients and oxygen to the biofilm surface [6]. It has also been proposed that biofilms respond to shear stress by regulating metabolic pathways and become stronger [22].

This unexpected result of the development of thick biofilms in turbulent flow might be attributed to several other factors. The change in the substrate flux is one of those factors. High shear stress conditions are found to cause a double effect on mass transfer properties; on one hand, turbulence facilitates high substrate diffusion in biofilms and on the other hand, the resulting denser biofilm reduces the diffusivity of substrate [11]. Another factor is the role of hydrodynamics in bacterial aggregation in the bulk water. Bacterial aggregation is an important biological process between bacteria under which they come together in the bulk water before they attach to the exposed surfaces as biofilms [23]. Evidence from freshwater samples [21, 24] suggests that there is a strong relationship between aggregation and flow regime. In the fluvial environment multi-species aggregates in the bulk flow have been shown to present distinct growth dynamics that are shear dependent [25].

Little is known regarding the initial colonisation of bacteria, which is a precursor to the formation of biofilms [26, 27], and how it is affected by the flow regime [10]. In the present study, the influence of different flow regimes was studied on biofilms grown for 4 weeks in drinking water with the hypothesis that turbulent flow is more likely to enhance their growth. Initial formation of biofilms was characterised as a function of the flow regime with the hypothesis that biofilms can be established on the surfaces even after a short time period of 10 h. Finally, the robustness of already established biofilms which were grown for 4 weeks under a constant flow regime was studied to changes to the flow regime, each of which lasted 24 h, assuming that biofilms would be able to respond to those changes.

From the engineering view, the study of flow conditions can contribute to the successful management and control of biofilm formation to avoid biocorrosion that leads to pipe material deterioration and subsequent reduced life of the pipe [28, 29]. Thus, this study was focused on the effect of different flow conditions on biofilm growth in drinking water with the objective to understand whether controlling the flow conditions could play an important role in the growth of biofilms in drinking water pipes. Specifically, turbulent flow was here hypothesised to be the flow regime under which biofilms could grow most in drinking water. Overall, this study will allow comparison of the initial biofilm formation, the development of biofilms and their structures between three very distinct flow regimes: the turbulent, transition and laminar. The knowledge that turbulence might promote biofilm formation in drinking water will be useful for water companies, which might search for ways to control the high shear and mixing conditions at specific parts of the drinking water distribution systems (DWDS).

## Materials and methods

### Reactor conditions

Biofilms were grown in a jacketed rotating annular reactor (model 1320 LJ, BioSurface Technologies, USA). The main advantage of this reactor is that the shear stress conditions can be easily controlled by its motor device, and the flow rate can be controlled independently of the shear stress. It has been suggested that reactors cannot accurately simulate the flow conditions of real drinking water distribution systems due to their geometry [30, 31]. However, the generic relationships between biofilms and flow regimes were the focus of this study and those flow regimes were confidently created in this reactor.

The reactor held 20 removable polycarbonate slides that were attached to its inner drum. The polycarbonate material was chosen as one of the plastic materials used in drinking water systems, which does not have a rough surface of corroded material [20, 32]. The jacket of the reactor allowed the temperature to be maintained in the system via heated water from a bath circulator (Isotemp Bath Circulator, Fisher Scientific, England, UK). The temperature was chosen at 16 °C as the representative temperature of DWDS in the United Kingdom for spring and summer [1]. The reactor was covered with aluminium foil to achieve dark conditions for biofilm growth.

This reactor was used to simulate flow conditions similar to those in a pipe with a radius equal to the gap between the two cylinders of the reactor, and mean velocity equal to the mean velocity of the reactor. The inner drum of the reactor was rotated at three different speeds to induce Taylor–Couette flows [33–35]; at 30 rpm (the Reynolds number, Re = 960 and the Taylor number, Ta = 233), which corresponds to laminar flow; at 57 rpm (Re = 1800 and Ta = 439), which corresponds to transition flow; at 217 rpm (Re = 6800 and Ta = 1682), which corresponds to turbulent flow. These three speeds of the reactor were used to simulate three different flow conditions in a pipe of 30.3 mm diameter with: average velocity of 0.03 m/s and shear stress at the wall of 0.007 N/m^2^ in laminar flow, average velocity of 0.07 m/s and shear stress at the wall of 0.02 N/m^2^ in transition flow, and average velocity of 0.25 m/s and shear stress at the wall of 0.07 N/m^2^ in turbulent flow [36–38].

The choice of the diameter of the pipe at 30.3 mm corresponds to the extremities of drinking water pipes where the service lines start [39–41]. In those parts of drinking water systems the control of flow conditions is very important as the disinfectant residual has been depleted and microbial activities are much higher than in the mains [42]. Additionally, the conditions in service lines are characterised by longer residence times, higher stagnation periods, reduced flow rates and higher temperatures compared to the mains [43].

### Reactor medium

The medium that the reactor was filled with consisted of 150 ml of nutrient medium and 850 ml of drinking water that was sampled from a domestic tap in Glasgow. The drinking water, which was used to feed the reactor, was taken from a tap that was fed from an internal plumbing system. The drinking water treatment plant, which supplies water to the location from which the water was sampled, uses surface water as its source. The treatment steps that are followed in this treatment plant are: coagulation, rapid gravity filtration, chlorine disinfection and orthophosphate dosing. The concentrations for mineral salts of the reactor medium were: ammonium sulphate (1.2 mg/l), ammonium chloride (0.9 mg/l), magnesium sulphate heptahydrate (0.3 mg/l), manganese chloride tetrahydrate (0.003 mg/l), copper sulphate pentahydrate (0.002 mg/l), cobalt sulphate heptahydrate (0.001 mg/l), sodium molybdate dehydrate (0.001 mg/l), zinc sulphate heptahydrate (0.01 mg/l), and boric acid (0.75 mg/l) [44], and the concentration for glucose of the reactor medium was 1.5 mg/l. These concentrations kept the bulk water conditions in the reactor oligotrophic and they were realistic for drinking water pipe flow conditions [2]. The compounds used here would probably increase slightly any chances for biofilm growth on the accessible surfaces of the reactor within a limited time period. They were used given that in real systems there are more favourable conditions for biofilm growth rather than in well-controlled reactor systems, which are sterilised at the onset of the experiments [1, 4, 7, 45].

The concentration of total chlorine of the drinking water, that was sampled from the tap, was measured immediately after its sampling using the USEPA DPD Method 8167 [46] and a colorimeter (DR 900 Hach Colorado, US) and was found at 0.36 mg/l. The total organic carbon of the reactor medium was monitored during the reactor operation using a TOC-L analyser (Shimadzu, Japan, Asia) as the difference between the total carbon and the total inorganic carbon and it was found to be 1.59 ± 0.88 mg/l. Finally, the concentration of cells and microcolonies in the bulk water of the reactor was determined using fluorescence microscopy and was found to be (5.8 ± 0.5) × 10^5^ cells/ml and (7.6 ± 0.1) × 10^3^ microcolonies/ml, respectively [47].

### Experimental processes

Three experiments were conducted, which are described here as “A”, “B” and “C” experiments. In each experiment, the same processes were followed. The first process, which is described here as “development”, is the process in which biofilm development was studied after 4 weeks of reactor operation (Table 1). During these 4 weeks, the reactor was operating under batch mode (zero flow rate) as a closed system to retain the biomass and ensure that biofilms would be established on its surfaces. In each experiment, the medium was manually added to the reactor and immediately after that, the reactor started to operate under the specific rotation speed associated with experiment A, B or C. The 10 out of the 20 slides of the reactor were sacrificed for sampling at the end of this process.

**Table 1:** Development process for A, B and C experiments

Name of experiment	Flow regime development process	Total time
		(weeks)
A	Turbulent	4
B	Transition	4
C	Laminar	4

The second process, which is described here as “formation”, is the process in which the biofilm initial formation was studied after 10 h (Table 2). The reactor was operating again under batch mode, without changing the reactor medium after the 4 weeks of reactor operation, under the same rotation speed as in the development process. Clearly during the 4 weeks that the biofilms became established there would have been “formation” of biofilms on the surfaces, but it was thought that this might be atypical formation because both the bulk water environment and the neighbouring surfaces would not have been conditioned by the presence of biofilms. In a real system, any new uncolonised surfaces would be placed into a distribution system where biofilms were already established up and downstream. It is for this reason that the formation process on clean slides was studied after the 4 weeks where biofilms had become established on the surfaces of the bioreactor. The reason to keep the reactor medium unchanged for the development and formation processes is to make sure that any changes in the growth and structure of biofilms are the consequence only of the flow conditions. The 10 slides that were sacrificed at the development process were replaced by 10 new and sterile slides at the start of the formation process. These were removed for analysis in pairs at 2-h intervals.

**Table 2:** Formation process for A, B and C experiments

Name of experiment	Flow regime formation process	Total time
		(h)
A	Turbulent	10
B	Transition	10
C	Laminar	10

The last process, which is described here as “changes of flow regime”, lasted 72 h for A and C experiments and 96 h for B experiment. The aim of this process was to test how the already established biofilms after the 4 weeks of the reactor operation responded to changes in the shear stress conditions each of which lasted only 24 h. During this process, the reactor was operating under recirculation mode. One litre of a similar medium that initially filled the reactor was recycled; the differences being that distilled water was used instead of drinking water and the recycling medium here was 10 times more diluted. This meant that bulk liquid initially contained few planktonic bacteria and the bacteria that did appear were primarily derived from eroding biofilms on the surfaces. That medium was recirculated with flow rate at 22 ml/min and retention time at 45 min, to minimise suspended cell growth and enhance biofilm growth on the reactor surfaces [4]. During this process, the rotation speed of reactor was changed every 24 h to switch to one of the three distinct flow regimes: turbulent, transition and laminar.

In Table 3 there is description of the changes in the flow regime that were imposed for each of the three experiments conducted. The rationale behind the decision about the flow regime in each 24-h interval was the following: the first flow regime was the same as the one in the development and formation processes. This allowed us to establish whether there were differences in biofilm growth due to the change of the mode of flow from batch to recirculation. After 24 h, the flow regime was ramped down for experiment A, ramped up for experiment C and set higher then lower for experiment B. Finally, for the changes of flow regime process the 10 slides that had remained in the reactor untouched from the start of each experiment were used.

**Table 3:** Changes of flow regime each of which lasted for 24 h for A, B and C experiments

Name of experiment	Flow regimes changes of flow regime process				Total time (h)
A	Turbulent	Transition	Laminar	N/a	72
B	Transition	Turbulent	Transition	Laminar	96
C	Laminar	Transition	Turbulent	N/a	72

### Gravimetric measurements

Three reactor slides were used to characterise the thickness and density of biofilms in each of the three experiments at the end of the development process [48]. In brief, the slides were removed from the reactor, drained for 5 min at a vertical position and weighed for the determination of the wet mass. Then, the slides were dried for 24 h at 65 °C in an oven and weighed again. After that, the dried biofilm was washed off the slides with distilled water and laboratory tissues. The clean slides were dried again for 24 h at 65 °C and then weighed again. The dry mass was determined by the weight difference of the slides with and without the dried biofilm. After calculating the biofilm thickness and volumetric biofilm density, the areal biofilm density was calculated as the product of the biofilm thickness and the volumetric biofilm density. Finally, gravimetric measurements were conducted prior to the onset of each of the three experiments, in which all sterile slides were weighed to determine their weight without any biofilm attached to them. For these measurements the Fisherbrand™ Analytical Balance MH-214 was used (Fisher Scientific, England, UK) with 0.0001 g precision.

### Microcolonies count measurements

Two slides were used to count the microcolonies on the reactor slides of each of the three experiments in each of the three processes. The biomaterial attached to each reactor slide was gently scraped from it using a sterile cell scraper (ThermoFisher Scientific, England, UK), and diluted in 5 ml distilled water. Then, the 5 ml samples were fixed with 0.5 ml of 2% formaldehyde [49] and filtered on Whatman® 0.2 µm membrane filters (Sigma-Aldrich, Irvine, UK). A solution of 1 ml of 10 µg/ml 4′, 6-diamidino-2-phenylindole (DAPI) (ThermoFisher Scientific, England, UK) was used to stain the microcolonies for 20 min in the dark. After that, the solution was filtered and then the membrane filter was dried and prepared for visualisation. Fields of view were obtained using the fluorescence microscope Olympus IX71 (Japan, Asia) with the UPlanFLN objective lens (Japan, Asia) with 10× magnification/0.30 numerical aperture. The filter used was the DAPI filter with excitation at 358 nm and emission at 461 nm. The microcolonies visualised had a diameter of approximately 10 µm and consisted of approximately 10 cells. Microscopic fields of view were obtained on each membrane filter to calculate the concentration of microcolonies so that the coefficient of variation of the measurements was less than 0.30 [47, 50, 51].

The concentration of cells on the reactor slides was also calculated following the same process as that described for the calculation of the concentration of microcolonies. The only difference is that after the biomaterial attached to the reactor slides was scraped from them, then it was homogenised in vortex for 2 min in 5 ml distilled water. Additionally, the membrane filters were covered with 1 ml of 0.1% Triton X-100 solution (ThermoFisher Scientific, England, UK) to evenly disperse the cells before DAPI staining was applied. Cells were finally visualised using the oil immersion UPlanFLN objective lens (Japan, Asia) with 100× magnification/1.30 numerical aperture and their concentration was calculated at (5.4 ± 0.6) × 10^5^ cells/cm^2^. Finally, the measurements for the calculation of the concentration of microcolonies and cells on the reactor slides were also conducted prior to the onset of each of the three experiments to ensure that the reactor slides were sterile.

### Biofilm structure measurements

For the biofilm structure measurements two slides were used for the development process, three slides were used for the formation process and three slides were used for the changes of flow regime process of each of the three experiments. The biofilms on the reactor slides were firstly fixed with 0.5 ml of 4% paraformaldehyde [52]. The samples were firstly covered with 1 ml of 10 µg/ml Fluorescein Aleuria aurantia lectin (Vector laboratories, England, UK) for 10 min in the dark to stain the lectin-specific EPS glycoconjugates, which consist the most part of the EPS [18, 20]. Then, the samples were covered with 1 ml of 10 µg/ml DAPI for 20 min in the dark to stain the cells. Biofilm structures were visualised using fluorescence microscopy with the oil immersion UPlanFLN objective lens with 100× magnification/1.30 numerical aperture.

The surface area of both cells and EPS glycoconjugates on the reactor surfaces was then calculated. Additionally, the surface area that only the EPS glycoconjugates occupied on the reactor surfaces was calculated. These surface areas were calculated in Matlab by processing the microscopic fields of view obtained. Again, the coefficient of variation of the measurements was less than 0.30. The composite microscopic images of biofilms (from the cells and EPS glycoconjugates) were created using the Matlab command called “imfuse”. The original microscopic images were firstly converted to grayscale images using the Matlab command called “rgb2gray” and then to binary images using the Matlab command called “im2bw” to separate the biomaterial (the biofilms in one case and only the EPS glycoconjugates in the other case) from the background of the microscopic image. After the surface area for all the microscopic images obtained was calculated, it was divided to the total surface area of the microscopic image to finally calculate the percentage of the surface area (%). The measurements for the calculation of the surface area of biofilms on the reactor slides were also conducted using phase contrast microscopy and the percentages of surface area of biofilms were found to be in agreement with those found from the images obtained using fluorescence microscopy that were described here.

### Statistical analysis

All measures were analysed in IBM SPSS Statistics using one of the following tests: (1) the one-way ANOVA test in conjunction with the Tukey’s and the Duncan-Waller’s tests, (2) the Kruskal–Wallis by ranks test, (3) the Jonckheere-Terpstra test and finally, (4) the Pearson’s chi-squared test in conjunction with the Phi and Cramer’s test, depending on the fitness of the data under comparison to the assumptions of the tests. All statistical calculations were based on the confidence level of 95%, which means that a *P* value lower than 0.05 was considered statistically significant.

Where the data sets were normally distributed and there was homogeneity of variances, then significant differences between data were tested using one-way ANOVA in conjunction with the Post hoc Tukey’s and Duncan-Waller’s tests that would further validate the statistical result of one-way ANOVA. Comparisons of the surface area of biofilms between the batch and recirculation mode of flow were tested using one-way ANOVA in conjunction with the Tukey’s and Duncan-Waller’s tests.

Where the data sets were not normally distributed, then the Kruskal–Wallis test was used, in which the variances of the populations should be equal across the samples. Comparisons of the surface area of biofilms between the different flow regimes for the changes of flow regime process were made using this test.

For the data in which the condition for the variances of the Kruskal–Wallis test was not met, the Jonckheere-Terpstra test was deployed, in which there should be a priori ordering of the populations. So, comparisons of the microcolonies that were attached to the reactor slides between the different flow regimes for the development process were made using this test. The same test was used for the comparisons of the surface area of EPS glycoconjugates between the different flow regimes for the formation process.

In the case in which the data sets did not validate the assumptions of any of the previous tests, the Pearson’s chi-squared test in conjunction with the Phi and Cramer’s test were performed for large size and independent samples.

### Spatial statistics

Textural entropy was one of the measures used in this study to characterise the biofilm structures. It is used to describe the randomness of the components of a grayscale image by comparing the intensity of the image pixels. The higher the value of the entropy, the more heterogeneous the biofilm is. This means that more complex biofilm structures are demonstrated in the image [53, 54]. Entropy was calculated using the Matlab function called “entropy”.

Semi-variograms were used here as another measure to characterise the spatial variance of biofilm structures within grayscale images and quantify the spatial dependencies in the data sets. Their function relates the semi-variance of the data points to the distance that separates them. Large distance of the data points means more data pairs for estimation of the semi-variance, but less amount of detail in the semi-variogram. In other words, semi-variograms are a way of graphically capturing the spatial variance of points on a landscape as a function of their distance. All combinations of points at a distance are collated and their variance is determined for all possible separation distances [55, 56].

An important part of a semi-variogram is the “origin”; the closest points of the diagram. Where values are co-located, for example, in clusters, the variances at short distances are low as the values are similar. At the characteristic cluster length, the variance will rapidly increase. Another important part of a semi-variogram is the “sill”; the variogram upper bound that is equal to the variance of the dataset and reflects the amount of variability. The sill is usually at large distances where there is no gradient in the diagram [57, 58]. In total, 12,000 points were used for the calculation of each semi-variogram. They were calculated using the Matlab function called “variogram.m” and they were created only for the most representative images obtained from fluorescence microscopy. These images were those in which entropy was equal to the average entropy of all the images obtained.

Autocorrelation function (ACF) diagrams were used as the last measure to characterise the biofilm structures. The ACF diagrams are, in essence, a two-dimensional extension of the semi-variograms. They allow us to assess how the spatial autocorrelation changes with distance. They correlate pixel intensities within grayscale images and detect the repetitive structures within the images under consideration by combining together all parts of them. The ACF diagrams are real-space images, so that their dimensions have the same meaning as in the original images [59].

In these diagrams, represented here as contour plots, the central element provides a measure of the size and shape of the basic element that dominates the original images. The rest of the contour lines reflect the size and shape of the neighbourhood elements of the original images. Finally, the bar on the right side of the diagrams provides a measure of the autocorrelation. The darker the colour of the bar, the less the autocorrelation value is with its lowest value to be zero and the highest value to be one [60]. The ACF diagrams were calculated using the Matlab function called “autocorr2d.m” and again created only for the most representative images.

## Results and discussion

### Development process

**Fig. 1:**
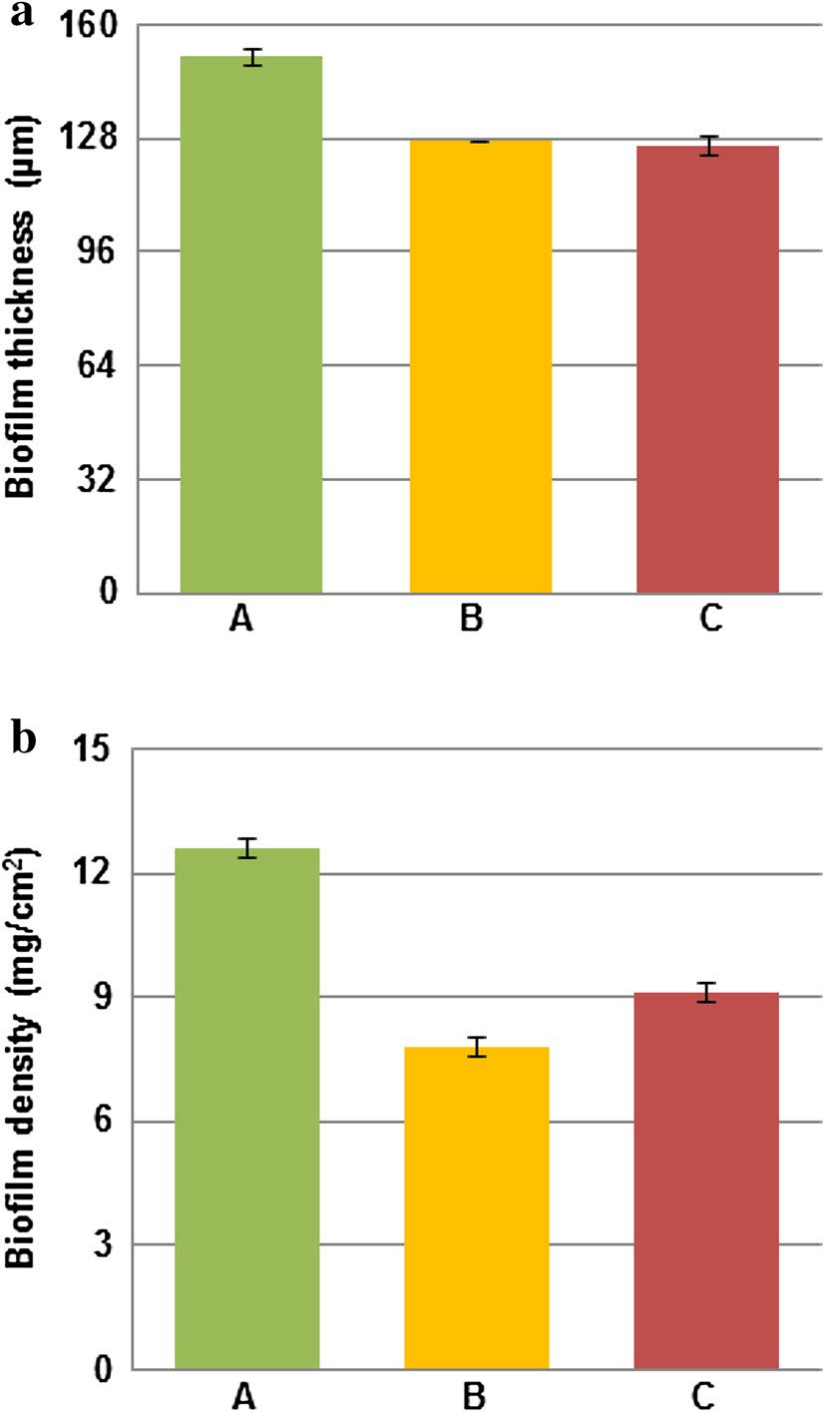
Biofilm thickness and density at the end of the development process. **a** Biofilm thickness (*n* = 6; mean with standard deviation) and **b** biofilm density (*n* = 6; mean with standard deviation). “A” describes turbulent flow, “B” describes transition flow and “C” describes laminar flow

**Fig. 2:**
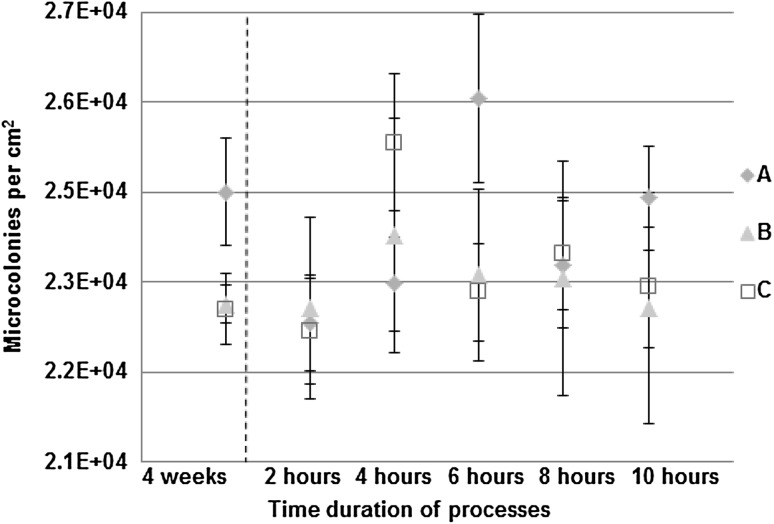
Concentration of microcolonies on the reactor surfaces (*n* = 60; mean with standard deviation). The dashed line indicates the move from the development to the formation process. “A” describes turbulent flow, “B” describes transition flow and “C” describes laminar flow

**Fig. 3:**
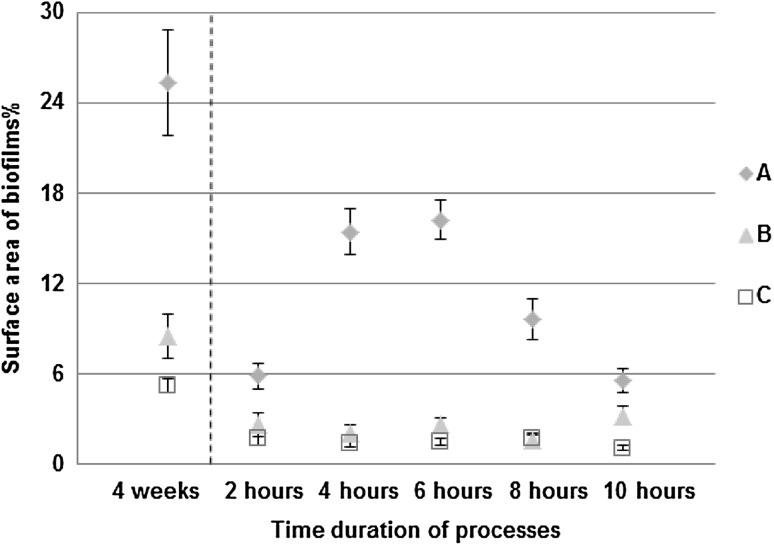
Percentage of surface area of biofilms (*n* = 60; mean with standard deviation). The dashed line indicates the move from the development to the formation process. “A” describes turbulent flow, “B” describes transition flow and “C” describes laminar flow

**Fig. 4:**
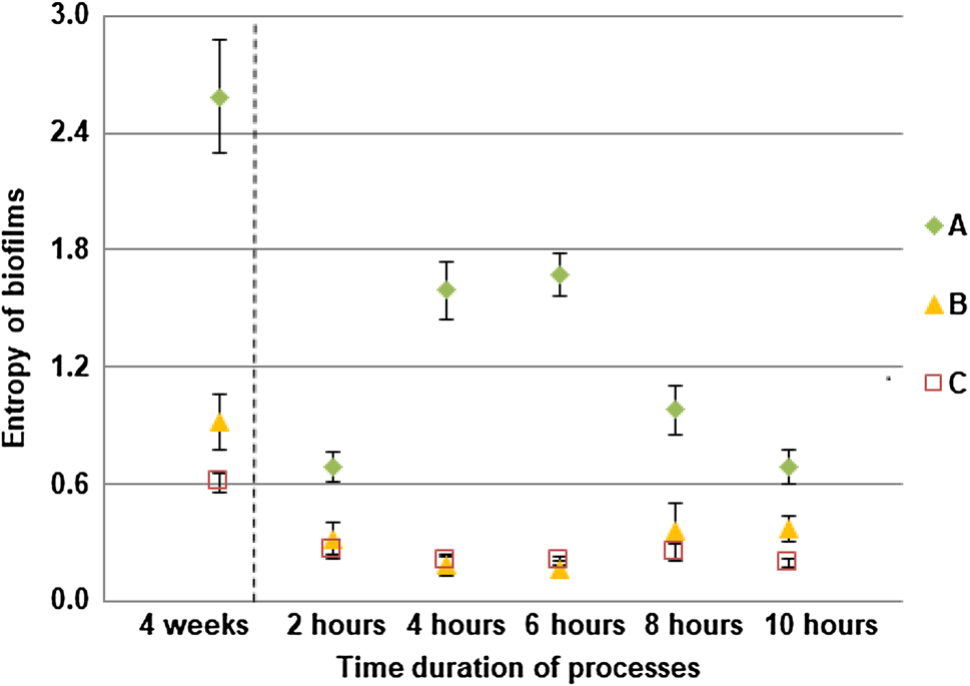
Entropy of biofilms (*n* = 60; mean with standard deviation). The dashed line indicates the move from the development to the formation process. “A” describes turbulent flow, “B” describes transition flow and “C” describes laminar flow

The biofilm thickness (Fig. 1a) and the biofilm density (Fig. 1b) were determined for all three experiments at the end of the development process. It was found that the highest thickness and density were for the biofilms developed in turbulent flow. Biofilms in drinking water are generally thin but the thicknesses that can be reached are variable. Thicknesses that have been recorded for biofilms in DWDS range from a few tens of micrometres [61] to a few hundreds of micrometres [62]. Biofilms may be formed on the surfaces within a few days or months and may reach a cell concentration of 10^7^–10^9^ cells/cm^2^ [5]. Additionally, at the end of the development process the number of microcolonies attached to the reactor slides (Fig. 2), the surface area of biofilms (Fig. 3) and the entropy of biofilms (Fig. 4) were found to be significantly higher (*P* < 0.05) in turbulent flow than in transition and laminar flow. No significant differences were found in these measures between the transition and laminar flow.

The measurements of the biofilm thickness and density, the concentration of microcolonies on the reactor surfaces, the percentage of surface area of biofilms and the entropy proved our hypothesis correct that turbulent flow was the flow regime in which biofilms were developed to a higher extent than the biofilms developed in transition and laminar flow. With the increase in shear stress associated with faster flows one might have expected biofilms to be smoother in the turbulent regime; where the biofilm protrudes into the flow the high stress could potentially shear off pieces of the biofilm eroding any lumps on the surface. However, this did not appear to be the case. The measure of roughness here is entropy and the entropy of biofilms was greatest in the turbulent regime. The increased number of microcolonies attached to the reactor slides and the increased growth of biofilms on the surfaces might mean that in turbulent flow more microcolonies actually come from the bulk liquid and land on the surfaces.

Drinking water distribution networks are designed for liquid velocities of about 0.2–0.5 m/s. Flow conditions can range from laminar to turbulent flow but stagnant waters also occur in places in which the water consumption is low [7]. In low-flow conditions, the transport of bacteria from the bulk water to the exposed surfaces occurs due to Brownian diffusion, sedimentation and cell motility. In high flow conditions, which are mostly experienced in such systems, microorganisms are transported by eddies in the flow [28, 63, 64]. Changes in the hydraulic conditions affect the quality of drinking water and thus, different pipes should be treated differently to obtain optimum operational effectiveness and minimise discoloration risk depending on their material composition [65]. Finally, the abundance, structure and composition of planktonic bacterial assemblages are also affected by the hydraulic conditions [66].

### Formation process

**Fig. 5:**
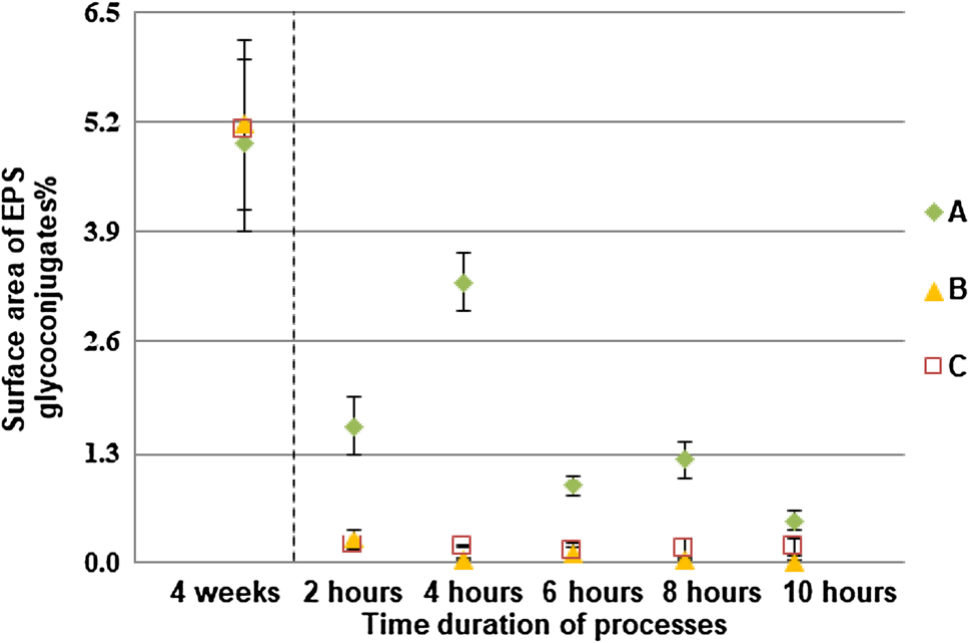
Percentage of surface area of EPS glycoconjugates (*n* = 60; mean with standard deviation). The dashed line indicates the move from the development to the formation process. “A” describes turbulent flow, “B” describes transition flow and “C” describes laminar flow

In the formation process, the surface area of biofilms (Fig. 3) and the entropy of biofilms (Fig. 4) were found to be significantly higher (*P* < 0.05) in turbulent flow compared to the surface area and the entropy found in transition and laminar flow, respectively. Again, no significant differences in these measures were found between the transition and laminar flow. The surface area of EPS glycoconjugates (Fig. 5) was found to be significantly higher (*P* < 0.05) in turbulent flow compared to transition and laminar flow in the formation process. Again, no significant differences were found in the surface area of EPS glycoconjugates between the transition and laminar flow.

It was shown that in turbulent flow the entropy and surface area of biofilms peaked at 6 h, and the surface area of EPS glycoconjugates peaked at 4 h. So, again turbulence was found to be critical in shaping the structure of the biofilm with area and roughness being consistently higher than in the other flow regimes. The peak in those measures might show that in the early stage of formation, microcolonies that were already rich in EPS glycoconjugates were deposited on the slides. As the deposits grew the surface area of biofilms increased and so did the entropy. With increased roughness and surface area of biofilms became increased and more heterogeneous shear stresses and thus, erosion served to decrease both the entropy and the surface area of biofilms and subsequently that of EPS glycoconjugates over the latter part of the 10-h formation period. These changes in the structure of biofilms for the different time periods of the formation process were significant only in turbulent flow. For the entropy and the surface area of biofilms, significant differences (*P* < 0.05) were found between 2–4, 2–6, 2–8, 4–8, 4–10, 6–8, 6–10 and 8–10 h. Finally, for the surface area of EPS glycoconjugates, significant differences (*P* < 0.05) were found between 2–4, 2–10, 4–6, 4–8 and 4–10 h.

### Changes of flow regime process

**Fig. 6:**
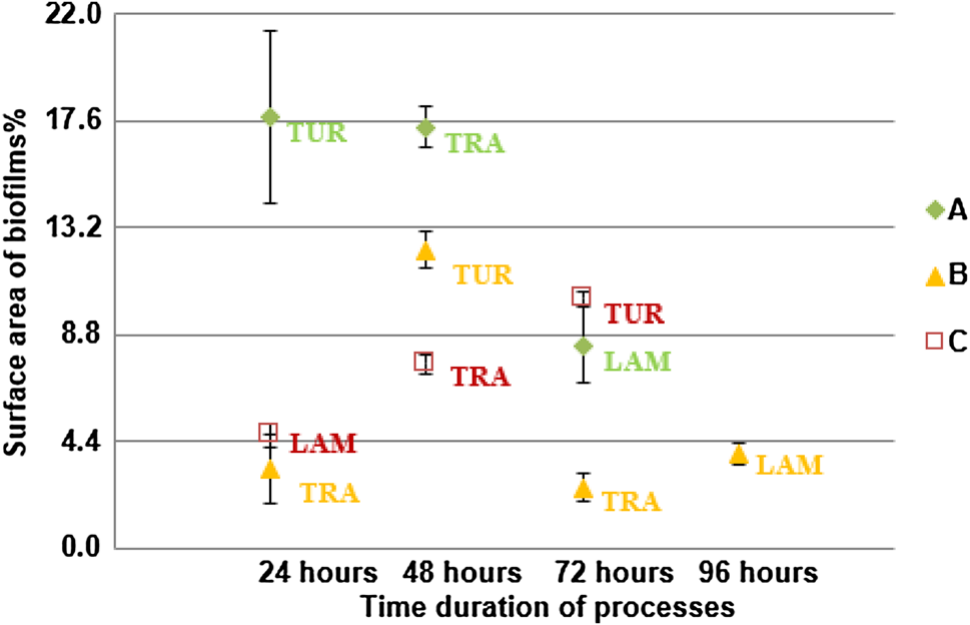
Percentage of surface area of biofilms in the changes of flow regime process (*n* = 60; mean with standard deviation). “TUR” describes the turbulent flow regime, “TRA” describes the transition flow regime and “LAM” describes the laminar flow regime in all three experiments (A, B & C)

**Fig. 7:**
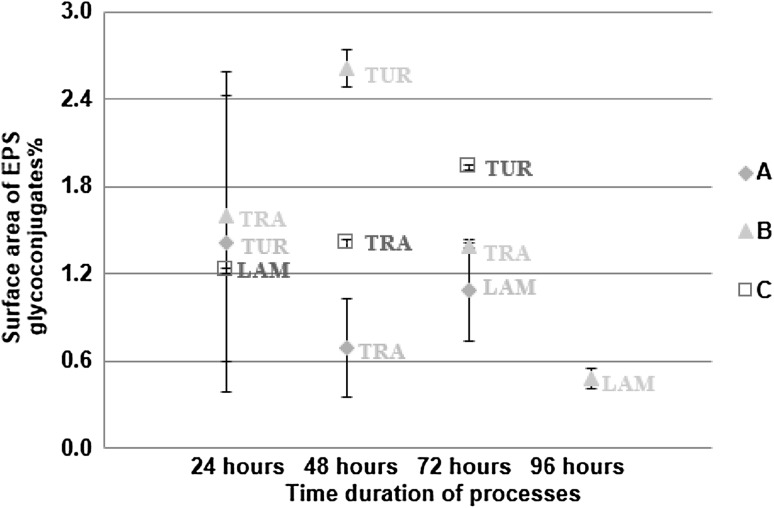
Percentage of surface area of EPS glycoconjugates in the changes of flow regime process (*n* = 60; mean with standard deviation). “TUR” describes the turbulent flow regime, “TRA” describes the transition flow regime and “LAM” describes the laminar flow regime in all three experiments (A, B & C)

In the changes of flow regime process, significant differences (*P* < 0.05) were found in the surface area of biofilms between the different flow regimes in each of the three experiments (Fig. 6). Additionally, significant differences (*P* < 0.05) were found in the surface area of EPS glycoconjugates between the different flow regimes in each of the three experiments (Fig. 7).

From the development process, which lasted 4 weeks, it was clear that the coverage of biofilms was increased with the flow speed. Additionally, when the bulk water was conditioned by the presence of biofilms, it was found that in the formation process the coverage of EPS glycoconjugates was increased with the flow speed. It was shown, from Figs. 6 and 7, that where rapid changes in the flow regime were applied even over a 24-h period, biofilms covered more surface area and produced more EPS glycoconjugates as the flow speed was increased. Consequently, it was shown that biofilms that were grown under a constant flow regime did not “forget” their characteristic morphology for that flow regime and responded to the 24-h changes to shear stress by converging to the characteristic morphology.

The formation of biofilms on the pipe walls is controlled by physical, biological and chemical processes [67]. One important biological process is the bacterial aggregation, in which microorganisms interact with each other forming a cluster that is free-floating and can be attached to a substratum as part-of or a precursor-to a biofilm [23]. This adhesion mechanism can influence the formation of complex multi-species biofilms in several diverse habitats [24]. Aggregation conveys many advantages to microorganisms in drinking water, such as enhanced transfer of chemical signals, exchange of genetic information, protection against harsh conditions and metabolic cooperation [68]. Evidence from the freshwater environment has shown that at higher shear rates, higher number of autoaggregating bacteria (same species) was found [21] than at lower shear stresses.

### Biofilm structures

**Fig. 8:**
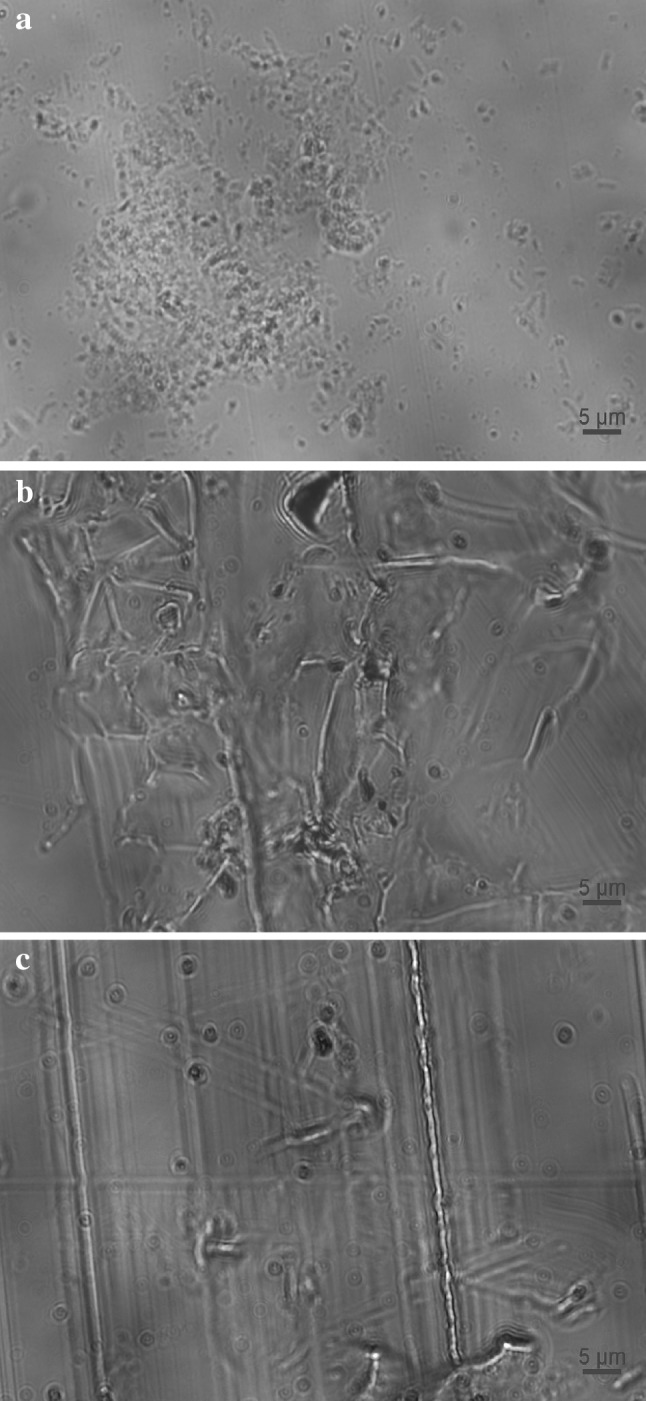
Biofilm structures at the end of the development process. **a** Patchy structures in turbulent flow, **b** linear structures in transition flow and **c** linear structures in laminar flow. The bar at the right bottom of the images indicates the distance in micrometres

**Fig. 9:**
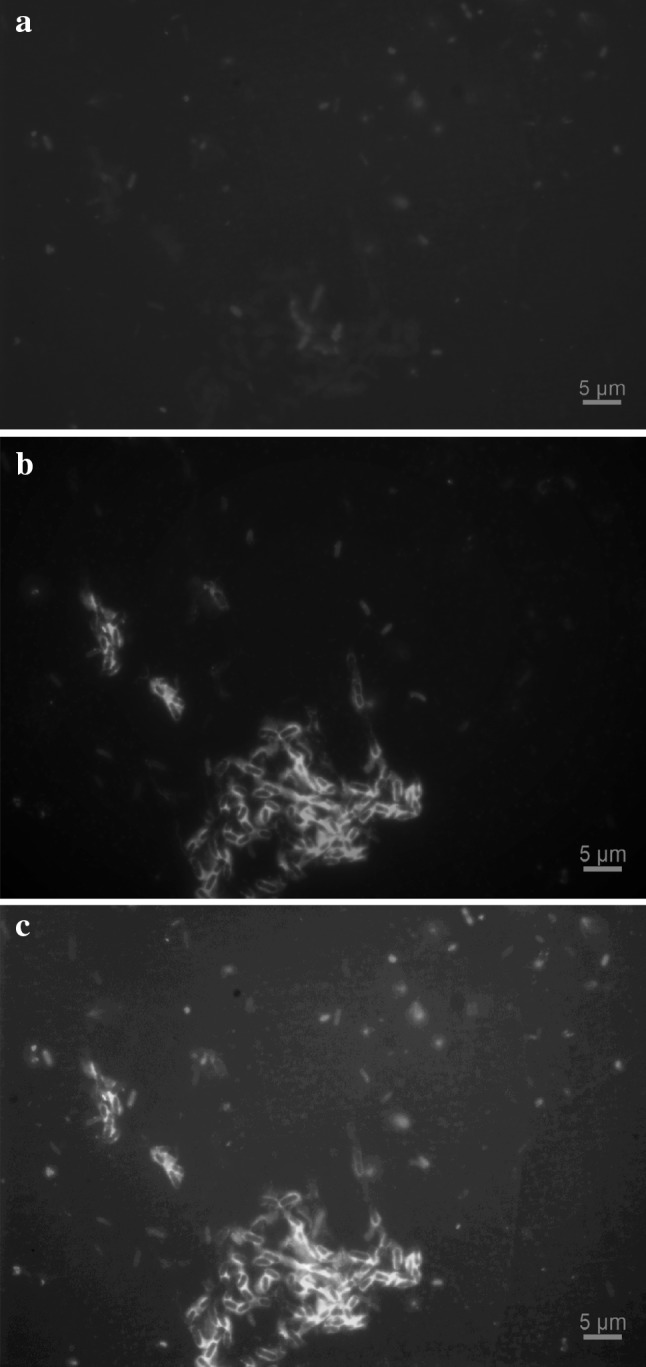
Biofilm structures at the end of the development process in turbulent flow. **a** Stained cells, **b** EPS glycoconjugates and **c** composite biofilm structure of both cells and EPS glycoconjugates. The bar at the right bottom of the images indicates the distance in micrometres

Biofilms were found to form either patchy structures consisting of microcolonies that are composed of densely packed cells held together by EPS in turbulent flow (experiment A) (Fig. 8a) or linear structures consisting of strands or streamers, possibly of EPS, with which the bacteria were associated in transition (experiment B) (Fig. 8b) and laminar flow (experiment C) (Fig. 8c) as revealed by fluorescence microscopy. Biofilms were also stained to reveal the cells (Fig. 9a) and EPS glycoconjugates (Fig. 9b) of their structures (Fig. 9c) using DAPI and Fluorescein Aleuria aurantia lectin, respectively. The composition of these patchy structures can generally range from a few cells to hundreds of micrometre-high cell clumps [69]. The formation of streamers, on the other hand, has been found to cause important effects on both the mass transport and oxygen distribution in the bulk liquid [70].

The exact structure of drinking water biofilms is still unclear and has not yet been described in detail due to difficulties in investigating such a small amount of biomass without disturbing it. This process is rendered even more complicated by the presence of debris, corrosion products and mineral deposits inside the pipe, which provide new niches for bacteria to colonise [2]. Organic and inorganic particles can accumulate in low-flow areas or dead-ends of DWDS and enhance microbial activities by providing protection for bacteria against harsh conditions. Any inorganic particles passing nearby may be incorporated in biofilms. There are inorganic particles such as sand that promote the erosion of biofilms whereas others such as clay may result in thicker and stronger biofilms [1, 71].

There are many types of biofilm heterogeneity [72–75]. Firstly, there is the geometrical heterogeneity (e.g., biofilm thickness, biofilm surface roughness, biofilm porosity, substratum surface coverage with biofilms). Secondly, there is the chemical heterogeneity (e.g., nutrients, metabolic products and inhibitors, pH variations, diversity of aerobic and anaerobic reactions). Thirdly, there is the biological heterogeneity (e.g., microbial diversity, activity of cells and EPS). Finally, there is the physical heterogeneity (e.g., biofilm density, biofilm strength, permeability, viscoelasticity, viscosity, EPS properties, solute concentration, solute diffusivity, presence of abiotic solids).

Biofilms are found to form very complex structures, which are influenced by many factors. Hydrodynamic conditions are one of the most significant factors affecting biofilm structures because they influence important variables, such as substrate loading rate and developing shear stresses [76]. The recent development of improved imaging techniques has allowed the visualisation of three-dimensional biofilm structures and spatial arrangement of different microbial species within them [17].

### Semi-variograms

**Fig. 10:**
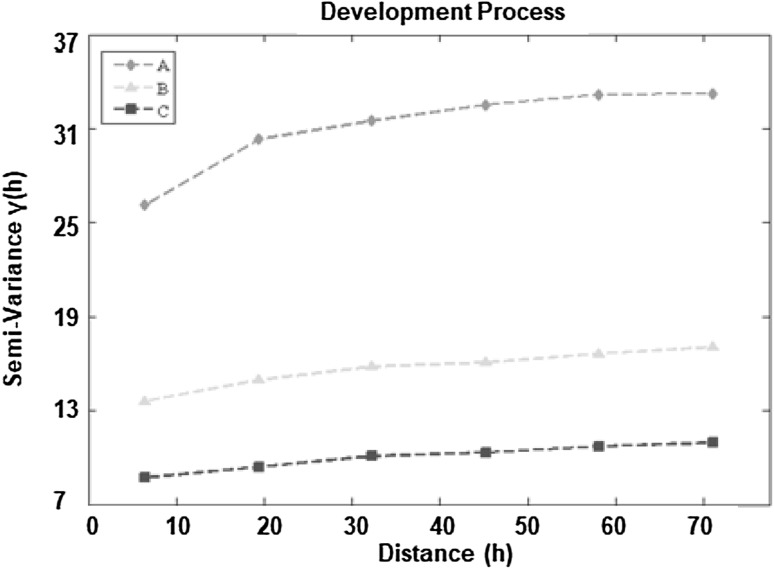
Semi-variograms in the biofilm development process (*n* = 12,000; mean). In the vertical axis is the semi-variance and in the horizontal axis is the distance for the calculation of the semi-variogram in micrometres. “A” describes turbulent flow, “B” describes transition flow and “C” describes laminar flow

The semi-variograms of the development process for all three experiments are here demonstrated (Fig. 10). The highest variance, represented by the sill of the semi-variograms, was found in turbulent flow and it was reached at about 60 microns. This merely suggests that there was a low degree of correlation between distant points on the biofilm surface topography, which is in agreement with the previous indication from the entropy measurements that the biofilm under turbulent flow conditions was the most heterogeneous.

The gradient in the variance close to the origin was the highest in turbulent flow. This shows that the topography of the biofilm was the most heterogeneous in turbulent flow. Specifically, the gradient in variance dropped after about 20 μm, which shows that there was a prevalence of topographic structures with a characteristic radius (or length scale) of approximately 20 μm. For the laminar and transition flows, the heterogeneity in the topography was much lower and there was a shallow linear gradient on the semi-variograms. This indicates a smoother surface potentially with features that extended over longer length scales than in the turbulent regime.

For the formation and changes of flow regimes processes, the data are changing rapidly so the semi-variograms are less informative since the overall variance fluctuated between time points in a way that made it difficult to draw any further conclusions. The semi-variograms in the formation and changes of flow regimes processes indicate broadly similar patterns with those in the development process. The biofilm irregularity was the highest in the development process, after biofilms were established on the reactor surfaces for 4 weeks, as the variance was found to be higher in the development process that in the other two processes.

### Autocorrelation function diagrams

**Fig. 11:**
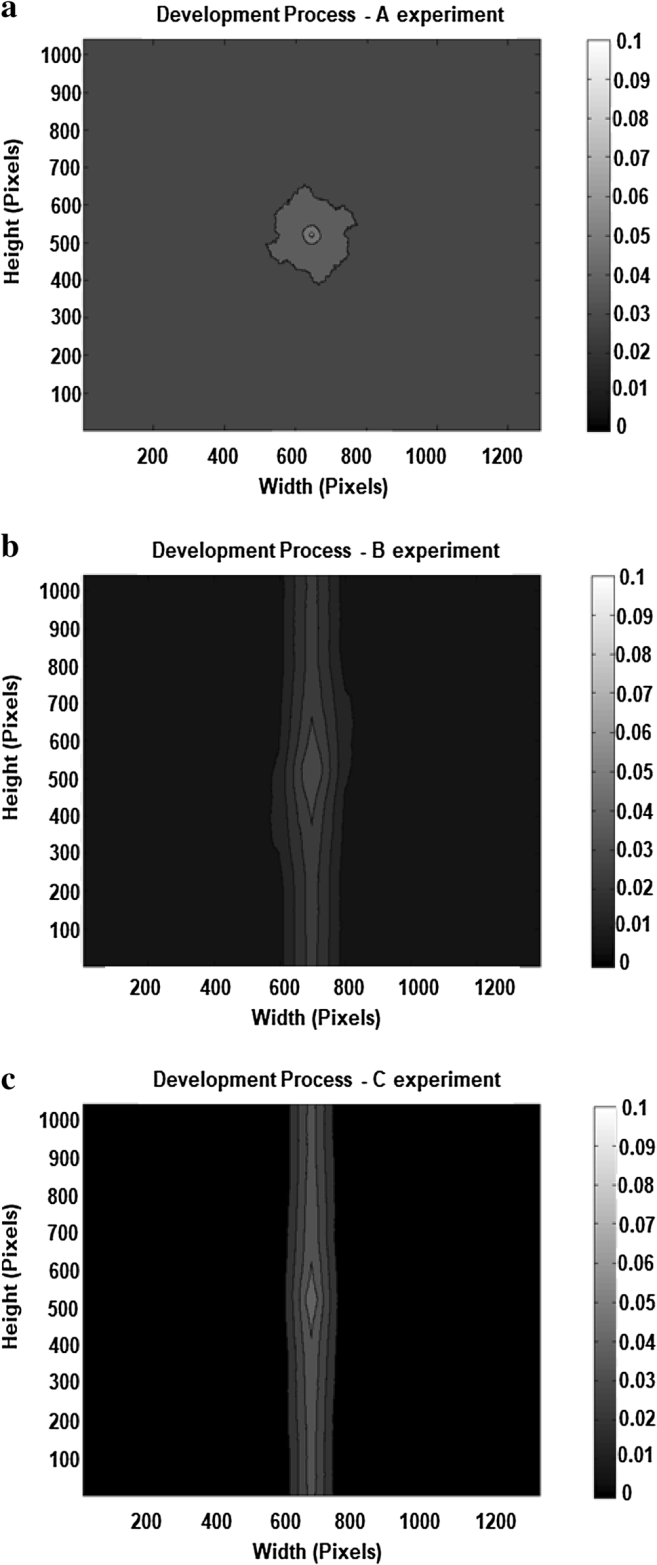
Autocorrelation function diagrams in the development process. **a** In A experiment, **b** in B experiment and **c** in C experiment. “A” describes turbulent flow, “B” describes transition flow and “C” describes laminar flow. The size in pixels of the contour plots is the same as of the original images based on which they were produced

The ACF diagrams of the development process for all three experiments are here demonstrated (Fig. 11). In Fig. 11a radially symmetric (circular) contours in autocorrelation are presented for turbulent flow conditions. This diagram is not suggesting that there is one spatially correlated “lump” at the centre of the image. It is the average autocorrelation for all pixels on the image. It demonstrates that on-average pixels are spatially correlated with their neighbours and this diminishes at about 100 pixels, which corresponds to approximately 10 μm. It suggests that radially symmetrical lumps are the prevalent topographical feature, which could be associated with microcolonies.

In transition (Fig. 11b) and laminar flow (Fig. 11c) there is a much higher degree of correlation at the direction perpendicular to the flow direction than along the flow direction. This indicates that the biofilm is arranged in linear structures that run perpendicular to the direction of flow. There does not seem to be any systematic regularity in the spacing between the linear features; had there been then the ACF diagram would have exhibited bands of higher correlation running parallel and either side of the main ACF feature. Finally, the highest degree of autocorrelation was found in turbulent flow, which suggests that in turbulent flow the biofilms consisted of cells that were piled up rather than dispersed as in the other two flow conditions.

The ACF diagrams are most useful in determining the structures that have been established over the development phase, rather than the rapidly changing formation or changes of flow regime processes of the three experiments. In both these processes, the only spatial structure identified for all three experiments was that of microcolonies. Again, the autocorrelation of biofilm structures was the highest in turbulent flow.

### Batch versus recirculation mode

Biofilms were studied for 4 weeks (development process) and for 10 h (formation process) while the reactor was operating under batch mode, which is not a typical condition for DWDS. The reason to do that was to emphasise the important differences that can be detected in biofilms under the different flow regimes in a closed well-controlled system. By the comparison between the batch mode at the end of 4 weeks of the development process and the recirculation mode at the end of the first 24 h of the changes of flow regimes process of each experiment, it was found that the surface area of biofilms, and the one of EPS were significantly higher (*P* < 0.05) in batch mode than in recirculation mode.

These differences might be explained by the fact that the surface area of the system was larger in the recirculation than in the batch mode. In the recirculation mode of flow, the system consisted not only of the reactor as in the batch mode, but also of the inlet and outlet polycarbonate pipes used to recycle the medium and the bottle in which the recycled medium was placed into. This means that biofilms could either be dispersed within the recycled medium or be attached to the additional surface area of the pipes. However, this was an unexpected result since the initial motivation to change the mode of flow in the changes of flow regime process was to favour the growth of biofilms with the extra recycled medium and the potential better recirculation of nutrients within the reactor.

Overall, within the DWDS between the two extremes of flow, the laminar one at the dead-ends of the service lines of DWDS and the turbulent one in the main part of DWDS, transition flow may occur [6, 77]. Therefore, this study was set up to look at how biofilms grow under the three distinct flow regimes: turbulent, transition and laminar flow. Understanding the impact of the flow regime on biofilms will be helpful in providing an insight on the functionality and mechanisms of biofilms during the early (hours) and moderate (weeks) stages of their life. This study will contribute to the consideration of future design of management strategies to control the flow conditions in real DWDS. Managing the flow, and especially changes in flow regime, will help in managing the density of microorganisms that finally appear at the tap. This bacterial density is related to biofilms that are formed on the inside surfaces of pipes as the vast majority of bacteria, which is estimated at 95% of the total cell population, are found to be attached to the surfaces, whereas, only 5% of the total cell population are found to be in the water phase [29, 78].

To effectively control biofilms by different strategies (i.e., process conditions and disinfection) it is essential for engineers to understand how they form and develop and the role that the flow conditions play on them. From this study it is clear that the control of flow conditions is very important for DWDS as it was indicated that in turbulent flow, which occurs in most engineered systems, biofilms form and develop to the highest extent. Thus, it is essential that we found a way to carefully control turbulent flow (perhaps by reducing the shear stresses) in parts of DWDS if we are to control or even prevent biofilm formation.
